# Supplementary material for: Experiences of receiving and providing maternity care during the COVID-19 pandemic in Australia: A five-cohort cross-sectional comparison
Source: PLoS One. 2021 Mar 24;16(3):e0248488. doi: 10.1371/journal.pone.0248488 (PMC7990294; doi:10.1371/journal.pone.0248488)
Supplement: S1 File — (DOCX) [file pone.0248488.s001.docx]

**S1 File. Experiences of Receiving or Providing Maternity Care during COVID-19 Pandemic in Australia**

Survey Items

Mapped, five cohorts

| **Comments/ Design** | **Women** | **Partners** | **Midwives** | **Medical Practitioners** | **Midwifery Students** |
| --- | --- | --- | --- | --- | --- |
| **SECTION 1**  **Demographics**  **KEY**  DD – Dropdown  FT – Free Text  RB – Radio Buttons | 1. State live in 2. Postcode live in 3. Are you of Aboriginal and/ or Torres Strait Islander origin? 4. Do you speak a language other than English at home? IF Y please describe 5. Country of birth – Australia (RB) – Other (Please specify) (FT) 6. Age groupings (5 year sets) (RB) 18 – 25, 26 – 30, 31 -35, 36 – 40, 41– 45, 46+ 7. Tested for C19? (DD)- once, twice, three or more times 8. Did any C19 tests return positive? Y/N | 1. State live in 2. Postcode live in 3. Are you of Aboriginal and / or Torres Strait Islander origin? 4. Do you speak a language other than English at home? IF Y please describe 5. Country of birth – Australia (RB) – Other (Please specify) (FT) 6. Gender Female, Male, Non Binary, Rather Not Say 7. Age groupings (5 year sets) RB 8. Tested for C19? DD- once, twice, three or more times 9. Did any C19 tests return positive? | 1. Australian State currently working in 2. Are you of Aboriginal and/ or Torres Strait Islander origin? 3. Do you speak a language other than English at home? IF Y please describe 4. Country of birth – Australia (RB) – Other (Please specify) (FT) 5. Gender Female, Male, Non Binary, Rather Not Say 6. Age groupings (5 year sets) RB 7. Tested for C19? DD- once, twice, three or more times 8. Did any C19 tests return positive? | 1. Australian State currently working in 2. Are you of Aboriginal and/ or Torres Strait Islander origin? 3. Do you speak a language other than English at home? IF Y please describe 4. Country of birth – Australia (RB) – Other (Please specify) (FT) 5. Gender Female, Male, Non Binary, Rather Not Say 6. Age groupings (5 year sets) RB 7. Tested for C19? DD- once, twice, three or more times 8. Did any C19 tests return positive? | 1. Australian State currently studying in 2. Are you of Aboriginal and/ or Torres Strait Islander origin? 3. Do you speak a language other than English at home? IF Y please describe 4. Country of birth – Australia (RB) – Other (Please specify) (FT) 5. Gender Female, Male, Non Binary, Rather Not Say 6. Age groupings (5 year sets) RB 7. Tested for C19? DD- once, twice, three or more times 8. Did any C19 tests return positive? |
| **SECTION 2**  **Shared questions** –  each cohort will answer a variation of the same question using  Agree – Disagree 6 point Likert continuum Strongly Agree, Agree, Somewhat Agree, Somewhat Disagree, Disagree, Strongly Disagree | 1. I feel/felt anxious about the impact of C19 on my wellbeing 2. I feel/felt anxious about the impact of C19 on the wellbeing of my family 3. I have been worried about exposure and potentially infecting myself and close family members with C19 because of my visits to the hospital 4. I feel/felt anxious about the (potential) impact of C19 on the wellbeing of my baby 5. I was happy with the changes to the way that maternity care is/was delivered at my health service during the C19 Pandemic 6. My expectations of my maternity care were met 7. I feel/ felt like I received timely and clear answers to my questions about the impact of C19 on me, my baby and family from my maternity care providers 8. The social distancing measures required during C19 mean/t that I feel / felt isolated from my maternity care providers 9. I am/was satisfied with the quality of care provided to me by my maternity care providers 10. I was happy with the way the health service was managing the risk of C19 11. Compared with my expectations, some of my experiences turned out better than I thought they would have during C19 12. Which 3 words best describe your experiences of receiving maternity care during the C19 pandemic [free text]   Thank you for your time, your participation in this survey is really appreciated. | 1. I feel/felt anxious about the impact of C19 on my wellbeing 2. I feel/felt anxious about the impact of C19 on the wellbeing of my family 3. I have been worried about exposure and potentially infecting myself and close family members with C19 because of my visits to the hospital. 4. I feel/felt anxious about the (potential) impact of C19 on the wellbeing of the baby 5. I was happy with the changes to the way that maternity care is/was delivered at my health service during the C19 Pandemic 6. My expectations of our maternity care were met 7. I feel/ felt like I received timely and clear answers to our questions about the impact of C19 on me, my baby and family from my caregivers 8. The social distancing measures required C19 mean/t that I feel / felt isolated from our caregivers 9. I am/was satisfied with the quality of care provided to me by our maternity care providers 10. I was happy with the way the health service was managing the risk of C19 11. Compared with my expectations, some of our experiences turned out better than I thought they might during C19 12. Which 3 words best describe your experiences of receiving maternity care during the C19 pandemic   Thank you for your time, your participation in this survey is really appreciated. | 1. I feel/felt anxious about the impact of C19 on my wellbeing 2. I feel/felt anxious about the impact of C19 on the wellbeing of my family 3. I have been worried about exposure and potentially infecting myself and close family members with C19 because of my work at the hospital. 4. I feel/felt anxious about the (potential) impact of C19 on the wellbeing of the in utero/ neonates in my care 5. I was happy with the changes to the way that maternity care is/was delivered at my health service during the C19 Pandemic 6. My professional expectations of providing maternity care during C19 Pandemic are being met 7. I feel able to provide timely and clear answers to questions about the impact of C19 on women and their families 8. The social distancing measures required due to C19 mean/t that I feel / felt isolated from women and their families 9. I am/was satisfied with the quality of care that I have been able to provide to women and their partners during C19 10. I was happy with the way the health service was managing the risk of C19 11. Compared with my expectations, some of my care experiences with women turned out better than I thought they might during C19 12. Which 3 words best describe your experiences of providing maternity care during the C19 pandemic   Thank you for your time, your participation in this survey is really appreciated. | 1. I feel/felt anxious about the impact of C19 on my wellbeing 2. I feel/felt anxious about the impact of C19 on the wellbeing of my family 3. I have been worried about exposure and potentially infecting myself and close family members with C19 because of my work at the hospital. 4. I feel/felt anxious about the (potential) impact of C19 on the wellbeing of the in utero/ neonates in my care 5. I was happy with the changes to the way that maternity care is/was delivered at my health service during the C19 Pandemic 6. My professional expectations of providing maternity care during C 19 Pandemic are being met 7. I feel able to provide timely and clear answers to questions about the impact of C19 on women and their families 8. The social distancing measures required due to C19 mean/t that I feel / felt isolated from women and their families 9. I am/was satisfied with the quality of care that I have been able to provide to women and their partners during C19 10. I was happy with the way the health service was managing the risk of C19 11. Compared with my expectations, some of my care experiences with women turned out better than I thought they might during C19 12. Which 3 words best describe your experiences of providing maternity care during the C19 pandemic   Thank you for your time, your participation in this survey is really appreciated. | 1. I feel/felt anxious about the impact of C19 on my wellbeing 2. I feel/felt anxious about the impact of C19 on the wellbeing of my family 3. I have been worried about exposure and potentially infecting myself and close family members with C19 because of my work at the hospital. 4. I feel/felt anxious about the (potential) impact of C19 on the wellbeing of the in utero/ neonates in my care 5. I was happy with the changes to the way that maternity care is/was delivered at my health service during the C19 Pandemic 6. My professional expectations of providing maternity care during C19 Pandemic are being met 7. I feel able to provide timely and clear answers to questions about the impact of C19 on women and their families 8. The social distancing measures required due to C19 mean/t that I feel / felt isolated from women and their families 9. I am/was satisfied with the quality of care that I have been able to provide to women and their partners during C19 10. I was happy with the way the health service was managing the risk of C19 11. Compared with my expectations, some of my care experiences with women turned out better than I thought they might during C19 12. Which 3 words best describe your experiences of providing maternity care during the C19 pandemic   Thank you for your time, your participation in this survey is really appreciated. |
